# Pain perception and patient satisfaction after conservative versus surgical low back pain management: Questionnaire-based comparative study

**DOI:** 10.6026/973206300221978

**Published:** 2026-04-30

**Authors:** Mohd Merazul Ashekin, Hemjit Das, Vidula Gowardhan, Mustafa AI-Jaafar

**Affiliations:** 1 Department of Internal Medicine, Aurora Specialized Hospital, Dhaka, Bangladesh; 2 Department of Orthopedics, Tinsukia Medical College & Hospital, Tinsukia, Assam, India; 3 Department of Pathology, NKP Salve Institute Medical Sciences and Research centre and Lata Mangeshkar Hospital Nagpur, India; 4 Department of Trauma and Orthopedics, Aneurin Bevan University Health Board, United Kingdom

**Keywords:** Low back pain (LBP), pain perception, conservative management, surgical management, patient satisfaction, questionnaire-based study, comparative analysis, treatment outcomes, rehabilitation, quality of life

## Abstract

Low back pain remains a leading cause of disability and uncertainty persists regarding optimal treatment selection between conservative and surgical approaches. A key reason that individuals have trouble deciding on the best route to take, surgical type or conservative type, as treatment options is because there is still a lack of clarity surrounding which type of treatment options to use. Therefore, it is of interest to analyse pain perception and patient satisfaction in 100 participants (50 patients receiving surgery, 50 patients receiving conservative treatment). Both conservative and surgical treatments resulted in significant reductions in pain scores; however, the surgical treatment group had a greater positive impact (mean pain reduction 68.9% versus 52.3%) and greater patient satisfaction (4.1 mean satisfaction score, versus 3.4 mean satisfaction score; p < 0.05). Functional improvement was greater following surgery and recurrence of back pain was more prevalent in the conservative treatment group. Thus, we show that the clinical severity, expected outcomes and patient preferences should be taken into consideration when selecting treatment options in order to optimize recovery and ensure patient satisfaction.

## Background:

Among musculoskeletal disorders, low back pain is the most common and the number one cause of disability all over the world [1]. Causes of low back pain vary; degeneration of discs, stenosis of the spinal canal and strains in the muscles are a few of many types of pain that may develop [2]. Chronic low back pain decreases a patient's ability to function physically as well as emotionally, creates barriers in regard to achieving an individual's potential at work and impacts their overall quality of life [3]. Depending on how severe the symptoms are as well as the degree of structural involvement with the spine, there are multiple types of treatments available: conservative management and surgical procedures [4]. In conservative therapy, the most common types of treatments include exercise therapy (physiotherapy), drugs and changes in lifestyle; surgical options are considered to be indicated only if conservative therapy fails to produce satisfactory results (refractory cases) or if the patient's low back pain is due to an anatomical defect [5]. Even with improvements in both conservative and surgical management, there are still questions about which treatment will provide better pain relief and about how satisfied patients will be with either type of management [6]. The results from a patient's own reports of how successful their treatment has been providing great insight into how well their treatment worked and should be considered along with conventional clinical evaluations of success [7]. Therefore, it is of interest to determine how patients perceive their pain and how satisfied they are with the management of their low back pain using both conservative and surgical interventions.

## Materials and Methods:

The participants in this study were patients diagnosed with lower back pain at a rehabilitation and orthopaedic centre. This was a questionnaire-based study that employed purposive sampling techniques to recruit patients with a total sample size of 100 Participants aged between 25 and 65 years, including 50 treated conservatively and the remaining 50 surgically. Patients diagnosed with malignancies/ infection/neurological disease were excluded. In addition, all healthcare professionals involved in conducting the research had obtained ethical approval and obtained informed consent from all participants. Demographic information about each participant was gathered via a validated structured questionnaire; while pain perception was assessed using the visual analog scale and patient satisfaction was evaluated via a likert-type system. After conducting expert review and pilot testing to establish questionnaire validation, the researchers used both descriptive and inferential statistical techniques to analyse data in order to compare mean pain scores and satisfaction levels between treated groups. A p-value of 0.05 or less was considered statistically significant for comparisons.

## Results:

A total of 100 patients suffering from low back pain were recruited equally into the conservative and surgical management groups, with the majority of patients aged between 46 and 55 years and slightly more males than females. Both conservative and surgical managements reduced the visual analogue scale (VAS) pain score; VAS mean scores for conservative managements reduced from 7.8 ± 1.2 to 3.9 ± 1.4 and for surgical managements reduced from 8.1 ± 1.1 to 2.5 ± 1.3. The percentage reduction of mean VAS score was significantly greater for surgical management (68.9%) than for conservative management (52.3%) (p < 0.001). Satisfaction scores were higher for surgical management patients (4.1 ± 0.7) than for conservative management patients (3.4 ± 0.8) (p = 0.002). Functional improvements were more often observed in post-operative patients; recurrences of pain were more prevalent in conservative management patients. Minor wound infections occurred only in patients who underwent surgical interventions. In conclusion, the results show that surgical management provides improved pain relief, greater functional improvement and better satisfaction compared to conservative management.

Table 1 (see PDF) shows the demographic characteristics of the study participants, indicating that the majority of patients were between 46 and 55 years of age, with a slightly higher proportion of males in both groups. Table 2 (see PDF) demonstrates the duration of low back pain before treatment, showing that most patients in both groups had symptoms lasting between 6 months and 2 years, with chronic cases more common among surgical patients. Table 3 (see PDF) compares pre-treatment and post-treatment pain perception using the visual analogue scale, revealing a significant reduction in pain scores in both groups, with greater improvement observed in surgically treated patients. Table 4 (see PDF) provides a detailed comparison of pain reduction categories, highlighting that marked improvement was more frequent in the surgical group than in the conservative group. Table 5 (see PDF) demonstrates the distribution of patient satisfaction levels, where a higher proportion of surgical patients reported being very satisfied compared to those receiving conservative management. Table 6 (see PDF) shows the extent of functional improvement after treatment, with surgical patients exhibiting higher rates of significant improvement in daily activities. Table 7 (see PDF) provides the correlation between pain reduction and satisfaction, indicating that higher pain relief was directly associated with greater satisfaction scores. Table 8 (see PDF) compares post-treatment complications, showing that recurrence of pain was more common in the conservative group, while minor wound infections were limited to surgical cases. Table 9 (see PDF) demonstrates patients' self-perceived overall treatment outcomes, reflecting better ratings in the surgical group. Finally, Table 10 (see PDF) compares the mean satisfaction and pain reduction scores between the two groups, confirming statistically significant superiority of surgical management in both parameters.

## Discussion:

The clinical effectiveness of surgical and conservative treatment approaches to managing low back pain has been demonstrated in this study by the direct comparative analysis of both approaches through the measurement of patient-reported metrics. Both treatment modalities had an equivalent positive impact in reducing the patient's pain and were confirmed as clinically effective [8]. However, the surgical approach was shown to provide a greater reduction in the patient's pain, increased satisfaction and an improved function compared to conservative treatment [9]. This corresponds with more recent findings on the topic that suggest that surgical management results in faster and larger reductions in symptoms in patients that present with structurally definable low back pathologies [1, 2]. For example, the average pain reduction of 68.9% achieved by the surgical group was significantly greater than the 52.3% average pain reduction observed in the conservative group. The level of satisfaction reported by patients with surgical management indicates a strong link between the quick achievement of a reduction in pain and the belief that the treatment has worked well [10s]. Additionally, the relationship between satisfaction and pain reduction serves to reinforce the need for the incorporation of patient- reported outcome measures (PROMs) into the ongoing evaluation of episodes of low back pain [3]. Although the conservative treatment was beneficial for patients, it should be considered a first line treatment option in those patients that experience mild to moderate symptoms [4]. After treatment, the conservative group experienced a higher incidence of recurrence than did the surgical group. Wounds classified as superficial or minor were noted only in patients who had undergone surgical management [11]. These findings represent a trade-off between non-invasive safety and the procedural risk associated with surgical treatment [12]. This study highlights the need to select patients for conservative or surgical treatment based on their presentation (i.e., severity, duration and structural pathology) [13, 14]. The incorporation of the patient's expectations and consideration of psychosocial aspects of their lives is believed to improve treatment outcomes [15]. The results of this study contribute to the existing literature by defining the differences between the satisfaction levels reported by patients and objective pain reduction levels as a balanced comparison of both surgical and conservative treatment strategies. Additionally, it emphasizes the need to evaluate treatment success from both an improvement in clinical status and the patient's perception of success, which further support the need to involve the patient in the decision-making process and develop individualized treatment algorithms for managing low back pain.

## Conclusion:

Surgical interventions appear to decrease pain levels and improve patient satisfaction more so than conservative care in that patient with low back pain appropriately selected for surgical treatment. Conservative care has a major role as the first-line treatment option however; conservative care typically produces a more gradual or less dramatic level of improvement than surgical interventions. In addition, the decision to perform a surgical procedure for low back pain should include consideration of clinical severity, structural (anatomical) findings and a patient's expectations regarding the outcome(s) of treatment. Hence, a treatment plan that includes these factors should result in the best possible and durable outcome for patients suffering from low back pain.
